# A TYPICAL WEEK WITH MILD COGNITIVE IMPAIRMENT: A PHOTO-ELICITATION STUDY

**DOI:** 10.1093/geroni/igz038.3125

**Published:** 2019-11-08

**Authors:** Jenny Wool, Brenna N Renn

**Affiliations:** 1 University of Washington, Seattle, Washington, United States; 2 University of Washington School of Medicine, Seattle, Washington, United States

## Abstract

Mild cognitive impairment (MCI) is an important precursor to dementia syndromes and carries with it both public and personal health significance, yet affected individuals may experience stigma, fear, and reluctance to participate in research or access services. Identifying the experience of people with MCI may help develop research agendas, interventions, and other supports to better match patients’ needs. To this end, we conducted photo-elicitation interviews with 11 community-dwelling adults aged 57-79 years with diagnosed MCI. Interviews took place remotely using teleconferencing software to reduce access barriers. Each semi-structured interview used 5-10 participant-generated photographs to elicit the experience of living with MCI, barriers to daily activities, and facilitators and supports. Interviews were transcribed, coded, and analyzed using Dedoose software. Qualitative analysis revealed themes of important activities, including physical activity, social engagement, and cognitive stimulation. Barriers presented by MCI included difficulty with former routines (e.g., cooking, finances), reduction of activities, and perceived stigma or fear of disclosure. Facilitators of daily activities included increased use of new strategies and environmental supports (e.g., calendars, smartphones), in addition to social and familial support. Multiple participants noted that the diagnosis of MCI led to opportunities for inner reflection and seeking a sense of inner calmness. Incorporating participant-generated images aided in data collection and facilitated discussion of sensitive topics with a cognitively impaired sample. Clinicians and researchers should support engagement in meaningful activities, assess barriers to important daily activities, and consider support to match the experience and needs of those with MCI.

